# Feeding practices of children within institution‐based care: A retrospective analysis of surveillance data

**DOI:** 10.1111/mcn.13352

**Published:** 2022-03-22

**Authors:** Emily DeLacey, Elizabeth Allen, Cally Tann, Nora Groce, Evan Hilberg, Michael Quiring, Tracy Kaplan, Tracey Smythe, Erin Kaui, Rachael Catt, Raeanne Miller, Maijargal Gombo, Hang Dam, Marko Kerac

**Affiliations:** ^1^ Department of Population Health, Faculty of Epidemiology and Population Health, London School of Hygiene & Tropical Medicine University of London London UK; ^2^ Nutrition and Health Services Holt International Eugene Oregon USA; ^3^ Centre for Maternal, Adolescent, Reproductive, & Child Health (MARCH), Department of Population Health, Faculty of Epidemiology and Population Health, London School of Hygiene & Tropical Medicine University of London London UK; ^4^ Department of Infectious Disease Epidemiology, Faculty of Epidemiology & Population Health, London School of Hygiene & Tropical Medicine University of London London UK; ^5^ MRC/UVRI & LSHTM Uganda Research Unit, London School of Hygiene & Tropical Medicine University of London Entebbe Uganda; ^6^ Department of Neonatal Medicine University College London Hospitals NHS Trust London UK; ^7^ UCL International Disability Research Centre, Department of Epidemiology and Public Health University College London London UK; ^8^ International Centre for Evidence in Disability, Department of Population Health, Faculty of Epidemiology and Population Health, London School of Hygiene & Tropical Medicine University of London London UK

**Keywords:** children, disability, epidemiology, feeding, institution‐based care, nutrition, orphanages

## Abstract

There is limited information on the feeding practices of 9.42 million children living within institution‐based care (IBC) worldwide. Poor feeding practices can predispose or exacerbate malnutrition, illness and disability. Here we describe the feeding practices of children living within IBC based on a retrospective analysis of records from 3335 children, 0–18 years old, participating in Holt International's Child Nutrition Program (CNP), from 36 sites in six countries. Data analysed included demographic information on age, sex, feeding practices, disabilities and feeding difficulties. Descriptive statistics were produced. A generalised linear model explored associations between feeding difficulties and disability and 2 × 2 tables examined feeding difficulties over time. An additional set of feeding observations with qualitative and quantitative data was analysed. At baseline, the median age of children was 16 months (0.66–68 months) with 1650/3335 (49.5%) females. There were 757/3335 (22.7%) children with disabilities; 550/984 (55.9%) were low birth weight; 311/784 (39.7%) were premature; 447/3113 (14.4%) had low body mass index and 378/3335 (11.3%) had feeding difficulties. The adjusted risk of having a feeding difficulty was 5.08 ([95% confidence interval: 2.65–9.7], *p* ≤ 0.001) times greater in children with disabilities than those without. Many children saw their feeding difficulties resolve after 1‐year in CNP, 54/163 (33.1%) for children with disabilities and 57/106 (53.8%) for those without disabilities. Suboptimal hygiene, dietary and feeding practices were reported. In conclusion, feeding difficulties were common in IBC, especially among children with disabilities. Supporting safe interactive mealtimes for children living within IBC should be prioritised, to ensure overall health and development.

## Key points

- Feeding difficulties are common among children living in institution‐based care (IBC), particularly but not exclusively among those children with disabilities.
- Suboptimal feeding practices were common in IBC and encompassed inadequate hygiene, limited support for self‐feeding, reading children's feeding cues (especially around pacing and satiety), addressing feeding difficulties, such as difficulty chewing or swallowing. These should be prioritised in training and supervision for caregivers.
- Addressing the needs of this vulnerable group should include support for safe feeding techniques. These should be prioritised to help ease the transition into eventual family‐based care if we are to move towards deinstitutionalizing children and strengthening families.

## Graphical abstract

Data cleaning flow chart of child health records data set

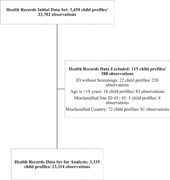

## 1 INTRODUCTION

It is estimated that anywhere from 3.18 million to 9.42 million children younger than 18 years old live in institution‐based care (IBC) globally (Desmond et al., 2020). IBC is defined by the United Nations as residential care provided in any nonfamily‐based group setting, such as places for emergency care and all other short‐ and long‐term residential care facilities (United Nations General Assembly, 2009; United Nations Human Rights Office of the High Commissioner, 1990). The UN Convention on the Rights of the Child requires that children in IBC are provided with standards of living, such as adequate nutrition, health care services and education, which support their full social integration and individual development (Richter et al., 2019; United Nations Human Rights Office of the High Commissioner, 1990). A focus on supporting children in IBC is important to ensure their full development. Substantial progress has been made in the last two decades in saving the lives of children younger than 5 years old globally (Victora et al., 2021). However, many children in IBC, especially those with disabilities have been excluded (DeLacey et al., 2020; Ernst et al., 2021). The UN Convention on the Rights of Persons with Disabilities defines persons with disabilities as ‘All persons with disabilities including those who have long‐term physical, mental, intellectual or sensory impairments which, in interaction with various attitudinal and environmental barriers, hinders their full and effective participation in society on an equal basis with others’ (United Nations, 2006).

These vulnerable children can be especially at risk for malnutrition. Malnutrition impacts millions of children worldwide who have limited access to nutritious food or the resources and support needed to safely and successfully eat. Nutritional intake is especially important throughout childhood because of critical periods of growth and development, during which unaddressed malnutrition can have long‐term consequences to children's development (Black et al., 2013; DeLacey et al., 2021; Yang, 2017). Feeding practices are an especially important factor in children's nutritional intake, and are defined as the interactions between a child and caregiver during mealtimes and can be influenced by various factors, such as socioeconomic status or a child's ability, age or cultural beliefs and practices (Reilly, 1996; B. N. S. Silva et al., 2017; Yang, 2017). Some children experience difficulty with feeding, impacting their ability to consume nutritious food. Feeding difficulties is a term that encompasses feeding issues or challenges, regardless of severity, aetiology or effects. It includes any difficulties that affect the process of providing food to the child or the child consuming the meal (Yang, 2017). Feeding difficulties affect up to 80% of children with disabilities and 25%–45% of those without (Benjasuwantep et al., 2013; Reif et al., 1995; Reilly et al., 1996; Yang, 2017). Feeding difficulties and malnutrition predispose children to long‐term impairments, such as diminished cognition, disability, suboptimal school performance and adult noncommunicable diseases (Black et al., 2013; UNICEF Producer, 2019; Victora et al., 2021).

Children in IBC, especially young children and those with disabilities, are particularly at risk for infections, illnesses, anaemia, micronutrient deficiencies and malnutrition (Black et al., 2013; DeLacey et al., 2020; DeLacey et al., 2021; The World Bank Group Producer, 2019; UNICEF Producer, 2019; Victora et al., 2021). A recent systematic review exploring the nutritional status of children living in IBC found few studies directly documenting the problem (DeLacey et al., 2020). One exemption, the St. Petersburg‐USA Orphanage Research Team found malnutrition in IBC related to inadequate dietary diversity; inappropriate types or textures of food or fluids; poor feeding and positioning practices; inadequate attention or stimulation and suboptimal hygiene and sanitation (The St. Petersburg‐USA Orphanage Research Team, 2005, 2008). These can result in increased frequency of illnesses or reduce nutrient utilisation (DeLacey et al., 2020; Frank et al., 1996; van IJzendoorn et al., 2011; The St. Petersburg‐USA Orphanage Research Team, 2008). The COVID‐19 pandemic threatens to exacerbate malnutrition in IBC for children already at risk due to their emotional, physical and social vulnerabilities (Goldman et al., 2020; Victora et al., 2021). This could include increasing their risk of social isolation or of immunodeficiencies, which make them more susceptible to COVID‐19 or even disruptions in food systems making nutritious food unavailable. (Goldman et al., 2020; Headey et al., 2020; Victora et al., 2021). Headey and coworkers suggest there could be a 14.3% increase in the prevalence of wasting among children younger than 5 years due to COVID‐19 (Headey et al., 2020). Concerns of increasing numbers of children being abandoned or separated from families due to COVID‐19 could lead to increased numbers in IBC (Goldman et al., 2020).

Children in IBC might be at risk for the following reasons. Firstly, facilities might only be able to address children's basic needs due to limited staffing, time and fiscal constraints (Frank et al., 1996; D. E. Johnson & Gunnar, 2011; The Children's Health Care Collaborative Study Group, 1994; Whetten et al., 2014). Often caregivers do not receive any information on developmental stages, caregiving, feeding practices or the needs of children of different ages or abilities (Richter et al., 2019; The St. Petersburg‐USA Orphanage Research Team, 2005). This is compounded by caregivers experiencing competing priorities for their time, resulting in interactions with children that are limited to routine and perfunctory caregiving (The St. Petersburg‐USA Orphanage Research Team, 2005, 2008). These competing priorities around mealtimes are of particular concern as feeding and mealtimes make up as much as 50% of the time a caregiver may spend with a child during the day and are key opportunities for interaction, learning and skill development (G. A. Silva et al., 2016; The St. Petersburg‐USA Orphanage Research Team, 2005). Additionally, caregivers are also responsible for other variables that impact feeding behaviour, such as sleep schedules, environment, activity time or access to appropriate feeding utensils and seating (Birch & Doub, 2014; The St. Petersburg‐USA Orphanage Research Team, 2005).

These challenges can be all the more severe for children with disabilities who comprise up to 25% of all children in IBC (DeLacey et al., 2020; DeLacey et al., 2021; Ernst et al., 2021). Disabilities are especially prevalent among children in low and middle‐income countries where IBC is common and malnutrition is the leading cause of childhood mortality (Black et al., 2013; Hume‐Nixon & Kuper, 2018; Victora et al., 2021). Children with disabilities often need additional time, support and assistance to safely, successfully and comfortably eat. With an estimated 93 million children (close to 1 in every 20 children worldwide) living with moderate to severe disabilities—this is an issue with far‐reaching implications (Groce et al., 2014; Hume‐Nixon & Kuper, 2018; Kuper et al., 2015; World Health Organization, 2011b). For some children, poor nutrition can also worsen their disabilities and make recovery more difficult if not impossible (Groce et al., 2014; Hume‐Nixon & Kuper, 2018; Victora et al., 2021).

This paper describes the current feeding practices of children living within IBC in a large multicountry nutrition programme. Our key objectives are to:

1. Describe the children's feeding methods, practices and associated difficulties.
2. Explore potential factors underlying these practices and difficulties, notably disability.
3. Explore any changes in feeding difficulties over time in IBC.

## 2 METHODS

### 2.1 Study design

This is a retrospective analysis of routine health records and programme audit data of feeding practices, dietary intake and feeding difficulties from a large multicountry IBC nutrition programme.

### 2.2 Setting/study size

Qualitative and quantitative data were collected from secondary data consisting of health records and routine programme audit behaviour observations of Holt International's Child Nutrition Program (CNP). Holt International is a nonprofit child welfare organization supporting children and families in multiple countries. Holt International's CNP is currently implemented in six countries: Vietnam, India, China, Mongolia, Philippines and Ethiopia. Within these countries, CNP is implemented in 53 community, foster care, day care and IBC sites, of which 36 IBC programmes were used for this study. Sample size was constrained by available programme data rather than determined by a priori calculation.

### 2.3 Participants and variables

Deidentified secondary data were used from the nutrition screening records of children aged 0–18 years old residing in IBC sites participating in the CNP. Nutrition screenings are routinely performed at each site. They are carried out monthly for children aged 0–2 years old; quarterly for those 2–5 years old and biannually thereafter. Each screening captures information on age, birth status, sex, disability status, episodes of illness, anthropometry, feeding methods and difficulties. Additionally, a smaller data set of deidentified feeding behaviour observations, completed by Holt's feeding experts during routine programme audits, were analysed from CNP baseline and evaluation reports. All included data are from January 2013 to May 2021.

### 2.4 Data management and analysis

Quantitative data were managed and analysed using Stata (16.1, StataCorp LLC). Data from each child's baseline and 1‐year screening were used for analysis. Children's records were provided by Holt International to the primary author (E. D.) in a deidentified CSV file. Data extracted from children's records included age, sex, prematurity, disability status, episodes of illness, anthropometry, feeding methods, dietary intake and feeding difficulties. Disability status was further grouped by the primary disability listed, as categorised by health professionals in the country. Low birth weight and preterm birth were added to children's records when available from any preadmission hospital records. However, birth status information was limited as many children were abandoned. Feeding variables included data on dietary intake, food supplements, feeding difficulties and vitamin/mineral supplementation. Different types of feeding difficulties were predefined by feeding specialists and could be recorded on a child's health record where present. Time since admission into IBC was a continuous variable defined as the number of days from the registered admission date to their exit date or to the date of the data export for those still in IBC. World Health Organization (WHO) diagnostic and data cleaning criteria were used based on age and gender thresholds for body mass index (BMI) and anaemia (World Health Organization, 2017, 2007, 2006). Haemoglobin levels for ages 0–5 years: mild 10.0–10.9 g/dl, moderate 7.0–9.9 g/dl, severe <7.0 g/dl; ages 5–11 years: mild 11.0–11.4 g/dl, moderate 8.0–10.9 g/dl, severe <8.0 g/dl; ages 12–14 years: mild 11.0–11.9 g/dl, moderate 8.0–10.9 g/dl, severe, <8.0 g/dl; females aged 14+ years: mild 11.0–11.9 g/dl, moderate 8.0–10.9 g/dl, severe <8.0 g/dl and males aged 14+ years: mild 11.0–12.9 g/dl, moderate 8.0–10.9 g/dl, severe <8.0 g/dl. BMI‐for‐age (BMIZ) outlier data cleaning cut‐offs: <−5 standard deviation (SD) and >+5 SD. *Z*‐score categories: risk of overweight/overweight: >+1 SD, normal weight: <+1 SD to >−2 SD, thinness/underweight: −2 SD to −3 SD, severe thinness/underweight: >−3 SD (World Health Organization, 2011a, 2007, 2006).

The smaller set of secondary routine programme audit data of behaviour observations of infant feeding, young child feeding and feeding of children with disabilities was completed by expert feeding specialists during baseline and evaluation assessments. These behaviour observations include quantitative and qualitative data. Quantitative data were from standard questions about specific practices; qualitative data were from comments on witnessed feeding practices, environment and hygiene practices. Qualitative data were managed and analysed using Microsoft Excel (2013).

### 2.5 Statistical methods/analysis

Descriptive statistics were produced for categorical and continuous variables. These are frequency and percent for categorical variables and mean (with SD) for normally distributed data, and median (with interquartile ranges [IQRs]) for nonnormally distributed data that were continuous variables.

The association between feeding difficulties and disability status was explored. For analysis of feeding difficulties over time, we cross‐tabulated those with and without feeding difficulties at 1‐year based on disability status and feeding difficulties at baseline. A generalised linear model with a log link was fitted to assess the association of feeding difficulties with disability status at children's baseline screening after adjusting for preidentified potential confounders age, and sex. Robust standard errors were used to allow for clustering by site. Statistical significance was taken as 5%.

For the quantitative data from behaviour observations, the frequency and percent of desired feeding behaviours at baseline and evaluation time points were calculated and then tested for nonrandom association using Fisher's exact test. Qualitative data from the feeding behaviour observations were summarised by grouping different comments into overarching themes (e.g., ‘child fed laying down’, ‘child fed with head back unable to safely swallow’, ‘child fed on lap without support and poor head positioning’) were all summarised as ‘inappropriate positioning’. The summary sought to identify categories and subcategories that appeared to be important in the experience and observation of feeding specialists. Themes were identified by most frequently recorded comments on observed practices. A narrative synthesis of findings was also undertaken.

## 3 RESULTS

### 3.1 Population demographics

**Figure 1:**
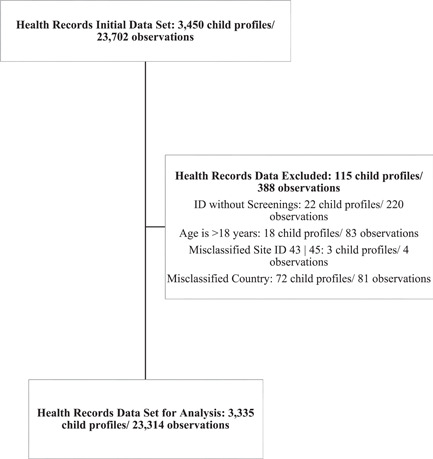
Data cleaning flow chart for health records data set

Figure 1 shows inclusion criteria leading to the final sample size. Table 1 shows baseline characteristics of all 3335 children living within IBC in six countries. There were many infants (0–6 months) (1041 [31.2%]) and children aged 5 years and older (1270 [38.1%]) in the programme. There were similar numbers of females and males. There were 757 (22.7%) children with one or more disabilities. Of these, cerebral palsy was the most commonly identified; however, in less than half (44.3%) of the children with a disability, the type of disability was not specified. Only 29.5% (985) of children had recorded birth information; among these low birthweight and prematurity were common, and both were more common among those with a disability when compared to those without (Table 1). Children entered into IBC at a median age of 16 months (IQR: 0.66–68 months) and stayed for a median time of 22.7 months (IQR: 8.8–48.8 months).

**Table 1:** Characteristics of children living within IBC in six countries at baseline screening

	All children	Children without disabilities	Children with disabilities
Population at baseline screening, *n* (%)	3335 (100.0)	2578 (77.3)	757 (22.7)
Total number exited	1795 (53.8)	1316 (51)	479 (63.3)
Active children	1540 (46.2)	1262 (49)	278 (36.7)
Exact date of birth unknown	3033 (90.9)	2344 (90.9)	689 (91)
Age based on the estimated or known date of birth			
0–6 months	1041 (31.2)	807 (31.3)	234 (30.9)
6–12 months	173 (5.2)	125 (4.9)	48 (6.3)
12–24 months	220 (6.6)	161 (6.3)	59 (7.8)
24–59 months	631 (18.9)	481 (18.7)	150 (19.8)
5–9 years	670 (20.1)	525 (20.4)	145 (19.2)
10–14 years	484 (14.5)	382 (14.8)	102 (13.5)
15–18 years	116 (3.5)	97 (3.8)	19 (2.5)
Sex: female, *n* (%)	1650 (49.5)	1306 (50.7)	344 (45.4)
Common disabilities, *n* (%)	‐	‐	n = 589
Autism spectrum disorder	‐	‐	12 (2.0)
Cerebral palsy	‐	‐	107 (18.2)
Cleft lip/cleft palate	‐	‐	8 (1.4)
Cognitive impairment	‐	‐	53 (9.0)
Down syndrome	‐	‐	21 (3.6)
Hearing loss/deafness	‐	‐	13 (2.2)
Heart disease/defect	‐	‐	43 (7.3)
HIV/AIDS	‐	‐	13 (2.2)
Hydrocephaly	‐	‐	16 (2.7)
Microcephaly	‐	‐	8 (1.4)
Vision impairment and blindness	‐	‐	23 (3.9)
Speech/language delays	‐	‐	6 (1.0)
Missing limbs/digits	‐	‐	3 (0.5)
Kidney disease or defect	‐	‐	2 (0.3)
Other	‐	‐	261 (44.3)
Birth weight unknown	2350 (70.5)	1878 (72.9)	472 (62.4)
Birth weight known, *n* (%)	*N* = 984	*N* = 699	*N* = 285
Birth weight > 2.5 kg	434 (44.1)	354 (50.6)	80 (28.1)
Low birth weight < 2.5 kg	452 (45.9)	305 (43.6)	147 (51.6)
Very low birth weight < 1.5 kg	81 (8.2)	33 (4.7)	48 (16.8)
Extremely low birth weight < 1.0 kg	17 (1.7)	7 (1.0)	10 (3.5)
Gestational age unknown, *n* (%)	2551 (76.5)	2042 (79.2)	509 (67.2)
Where birth prematurity status known, *n* (%)	*N* = 784	*N* = 536	*N* = 248
Full term	473 (60.3)	400 (74.6)	73 (29.4)
Premature	311 (39.7)	136 (25.4)	175 (70.6)
Median age (IQR) (months)	*N* = 3315	*N* = 2562	*N* = 753
	16 (0.66−68)	22.7 (0.66−72.5)	6.7 (0.7−48)
Median time since admission into IBC (IQR) (months)	*N* = 3209	*N* = 2499	*N* = 710
	22.7 (8.8− 48.8)	20.1 (7.9−40.7)	36.3 (15.6−75.8)

Abbreviations: IBC, institution‐based care; IQR, interquartile range.

### 3.2 Feeding and health characteristics

Table 2 describes feeding characteristics of all children at their baseline and 1‐year screening by disability status. See Table A1 for fuller details. With regard to feeding characteristics, feeding difficulties were common especially for children with disabilities. For those with feeding difficulties, the most common were difficulty feeding self for children older than 1 year, poor appetite and difficulty chewing. Of the total population at baseline, 225/3335 (6.8%) were taking food supplements and 1626/3335 (48.8%) were taking vitamin or mineral supplements, of which vitamin C, D, calcium and iron were the most common. Cough or colds were the most common illnesses experienced by children in the month before their last screening. Anaemia was prevalent at baseline (763/2828 [27%]) and at 1 year (97/1511 [6.4%]) and more prevalent among children with disabilities at both time points. At baseline, 447/3113 (14.4%) of children had low BMI.

**Table 2:** Description of feeding practices and health variables of children living within institution‐based care in six countries at baseline and 1‐year screening

Feeding profile	All children at baseline	All children at 1 year	Children without disabilities at baseline	Children without disabilities at 1 year	Children with disabilities at baseline	Children with disabilities at 1 year
Feeding method, *n* (%)^a^	*N* = 3335	*N* = 1909	*N* = 2578	*N* = 1385	*N* = 757	*N* = 524
Fed with bottle	1398 (41.9)	525 (27.5)	1028 (39.9)	327 (23.6)	370 (48.9)	198 (37.8)
Self‐fed^b^	1727 (51.8)	1046 (54.8)	1469 (57)	830 (59.9)	258 (34.1)	216 (41.2)
Fed with cup	930 (27.9)	650 (34.1)	804 (31.2)	504 (36.4)	126 (16.6)	146 (27.9)
Spoon fed	1123 (33.7)	957 (50.1)	811 (31.5)	628 (45.3)	312 (41.2)	329 (62.8)
Fed with adaptive utensils	32 (1.0)	21 (1.1)	15 (.6)	2 (0.1)	17 (2.3)	19 (3.6)
Breastfed	9 (0.3)	1 (0.1)	7 (0.3)	1 (0.1)	2 (0.3)	0
Feed type, *n* (%)^a^						
Formula	1488 (44.6)	467 (24.5)	1108 (43)	316 (22.8)	380 (50.2)	151 (28.8)
Solid foods	1993 (59.8)	1314 (68.8)	1578 (61.2)	951 (68.7)	415 (54.8)	363 (69.3)
Animal milk	803 (24.1)	584 (30.6)	659 (25.6)	402 (29.0)	144 (19.0)	182 (34.7)
Rice cereal	445 (13.3)	534 (28)	306 (11.9)	339 (24.5)	139 (18.4)	195 (37.2)
Breast milk	11 (0.3)	1 (0.1)	9 (0.4)	1 (0.1)	2 (0.3)	0
Special diet^c^	77 (2.3)	53 (2.8)	38 (1.5)	19 (1.4)	39 (5.2)	34 (6.5)
Feeding difficulty, *n* (%)						
Feeding issue present	378 (11.3)	243 (12.7)	153 (5.9)	83 (6.0)	225 (29.7)	160 (30.5)
Aspiration	14 (0.4)	11 (0.6)	0	2 (0.1)	14 (1.9)	9 (1.7)
Difficulty sucking	27 (0.8)	16 (0.8)	4 (0.2)	0	23 (3.0)	16 (3.1)
Cough/chokes during feeding	57 (1.7)	25 (1.3)	17 (0.7)	0	40 (5.3)	25 (4.8)
Difficulty feeding self (>1 year)	119 (3.6)	103 (5.4)	8 (0.3)	18 (1.3)	111 (14.7)	85 (16.2)
Reflux/heartburn	6 (0.2)	5 (0.3)	2 (0.1)	0	4 (0.5)	5 (1.0)
Poor appetite	111 (3.3)	82 (4.3)	72 (2.8)	47 (3.4)	39 (5.2)	35 (6.7)
Frequent vomiting/spitting up	19 (0.6)	8 (0.4)	7 (0.3)	1 (0.1)	12 (1.6)	7 (1.3)
Difficulty drinking from a cup (> 1 year)	53 (1.6)	43 (2.3)	2 (0.1)	3 (0.2)	51 (6.7)	40 (7.6)
Difficulty swallowing	63 (1.9)	50 (2.6)	3 (0.1)	0	60 (7.9)	50 (9.5)
Difficulty chewing	91 (2.7)	82 (4.3)	8 (0.3)	1 (0.1)	83 (11.0)	81 (15.5)
Picky eater	69 (2.1)	44 (2.3)	35 (1.4)	19 (1.4)	34 (4.5)	25 (4.8)
Food allergy/intolerance	14 (0.4)	8 (0.4)	10 (0.4)	5 (0.4)	4 (0.5)	3 (0.6)
Bad teeth (> 1 year)	22 (0.7)	24 (1.3)	9 (0.4)	4 (0.3)	13 (1.7)	20 (3.8)
Other	5 (0.2)	1 (0.1)	2 (0.1)	0	3 (0.4)	1 (0.2)
Supplements, *n* (%)^a^						
Currently taking food supplements	225 (6.8)	42 (2.2)	157 (6.1)	15 (1.1)	68 (9.0)	27 (5.2)
Currently taking mineral/vitamin supplements	1626 (48.8)	847 (44.4)	1176 (45.6)	572 (41.3)	450 (59.5)	275 (52.5)
Illnesses/symptoms, *n* (%)^a^						
Fever	438 (13.1)	193 (10.1)	295 (11.4)	121 (8.7)	143 (18.9)	72 (13.7)
Constipation	40 (1.2)	13 (0.7)	22 (0.9)	0	18 (2.4)	13(2.5)
Diarrhoea	172 (5.2)	40 (2.1)	116 (4.5)	23 (1.7)	56 (7.4)	17 (3.2)
Nausea/vomiting	163 (4.9)	32 (1.7)	111 (4.3)	20 (1.5)	52 (6.9)	12 (2.3)
Cough/cold	722 (21.6)	395 (20.7)	489 (19.0)	222 (16.2)	233 (30.8)	173 (33.0)
Hospitalisation	135 (4.0)	45 (2.4)	64 (2.5)	26 (1.9)	71 (9.4)	19 (3.6)
Anaemia status, *n* (%)	*N* = 2828	*N* = 1511	*N* = 2167	*N* = 1101	*N* = 661	*N* = 410
None	2065 (73.0)	1314 (87.0)	1604 (74.0)	969 (88.0)	461 (69.7)	345 (84.2)
Mild	438 (15.5)	136 (9.0)	346 (16.0)	102 (9.3)	92 (13.9)	34 (8.3)
Moderate	307 (10.9)	59 (3.9)	212 (9.8)	30 (2.7)	95 (14.4)	29 (7.1)
Severe	18 (0.6)	2 (0.1)	5 (0.2)	0	13 (2.0)	2 (0.5)
Body mass index for age *z*‐score *n* (%)	*N* = 3113	*N* = 1790	*N* = 2408	*N* = 1312	*N* = 705	*N* = 478
Overweight (>+1 SD)	361 (11.6)	226 (12.6)	286 (11.9)	172 (13.1)	75 (10.6)	54 (11.3)
Normal weight (−2 to +1 SD)	2305 (74.0)	1404 (78.4)	1882 (78.2)	1051 (80.1)	423 (60.0)	353 (73.9)
Underweight (<−2 to −3 SD)	291 (9.4)	119 (6.7)	175 (7.3)	73 (5.6)	116 (16.5)	46 (9.6)
Severely underweight (<−3 to ≥−5 SD)	156 (5.0)	41 (2.3)	65 (2.7)	16 (1.2)	91 (12.9)	25 (5.2)

^a^ Not mutually exclusive variables.

^b^ Self‐fed/self‐feeding is defined as when children feed themselves using their own fingers, utensils and cups. It is the process of setting up, arranging and bringing food and liquid from a plate, bowl or cup to their mouth. Self‐feeding using the fingers typically begins around 6–7 months old when children start eating solid foods. Typically by 12–14 months old, children take on more of an active role using spoons and cups on their own to feed themselves. Age‐appropriate self‐feeding is considered an important developmental skill (Holt International; Kaplan, 2019).

^c^ Special diets include diets for certain food allergies/intolerances or chronic conditions, such as diabetes, epilepsy or kidney disease. They also include therapeutic diets, such as modified texture diets like pureed, soft or liquid diets.

### 3.3 Feeding difficulties over time

Table 3 shows the change in feeding difficulties after 1 year in the CNP for those with and without disabilities. For those with a disability and no feeding difficulties at baseline, 279/315 (88.6%) continue to not have feeding difficulties and 36/315 (11.3%) develop feeding difficulties after 1 year. For those children with a disability and a feeding difficulty at baseline, 54/163 (33.1%) see their feeding difficulties resolve and 109/163 (66.9%) continue to have feeding difficulties.

**Table 3:** 2 × 2 tables of the change in feeding difficulties after 1 year in the CNP for those with and without disabilities

	Without feeding difficulties at 1 year	With feeding difficulties at 1 year	Total
With disabilities	333 (69.7%)	145 (30.3%)	478 (100%)
Without feeding difficulties at baseline	279 (88.6%)	36 (11.3%)	315 (100%)
With feeding difficulties at baseline	54 (33.1%)	109 (66.9%)	163 (100%)
Without disabilities	1333 (93.2%)	98 (6.9%)	1431 (100%)
Without feeding difficulties at baseline	1276 (96.3%)	49 (3.7%)	1325 (100%)
With feeding difficulties at baseline	57 (53.8%)	49 (46.2%)	106 (100%)

*Note*: Missing data excluded.

For children without disabilities and without feeding difficulties at baseline, after 1 year 1276/1325 (96.3%) continue to not have a feeding difficulty and 49/1325 (3.7%) develop a feeding difficulty. For those without disabilities and with feeding difficulties at baseline, 57/106 (53.8%) see their feeding difficulties resolve and 49/106 (46.2%) see their feeding difficulties continue after 1 year in the CNP.

### 3.4 Feeding difficulties and disability status

At baseline, 153/2578 (5.9%) children without disabilities had a feeding difficulty present; in contrast, 225/757 (29.7%) of children with a disability had feeding difficulties at baseline. A generalised linear model with a log link was fitted to explore the association between disability at baseline and feeding difficulties at baseline. We found an adjusted risk ratio of 5.08 (95% confidence interval [CI]: 2.65–9.7, *p* ≤ 0.001). This represents significantly increased risk of having a feeding difficulty among those with disabilities.

### 3.5 Feeding and positioning behaviour observations

Table 4 summarises the positioning and feeding behaviour observations for children with disabilities. From baseline to evaluation, a change was observed in behaviours of children receiving modified liquid or food textures. Additionally, observations of appropriately sized spoons or food offerings indicated a significant difference from baseline to evaluation. Observations indicate that meals frequently did not include all five food groups, handwashing was often skipped, children did not feed themselves and were often incorrectly positioned for mealtimes. Positive caregiver interaction with the child during meal times was also observed, such as smiling and making eye contact with children. Suboptimal feeding practices, poor hygiene practices, inadequate fluid and dietary intake were commonly observed. Putting cereal in formula bottles with cut nipples for children of all ages was noted. Further details are in Tables A2 and A3 on infant and young child feeding behaviour observations.

**Table 4:** Feeding and positioning behaviour observations for children with disabilities at baseline and evaluation

Feeding and positioning behaviour observations for children with disabilities				
	22 observations	29 observations	Fisher's exact (two‐sided) *p*‐value	Qualitative summary
	Baseline *n*/*N* (%)	Evaluation *n*/*N* (%)		Length of feeding: 5−60 min
Child's body positioned upright and feet supported	5/17 (29.4)	17/27 (62.9)	0.062	Inappropriate positioning; limited handwashing observed; limited or no fluids offered; limited self‐feeding; inappropriate feeding utensils; inadequate interaction; inadequate dietary intake; food textures are modified; children fed only on a schedule; hunger cues are not observed; fast pacing of meals; caregivers are attentive to children; environments were calm, quiet and appropriately lit
Child's hands cleaned before mealtime	0/9 (0)	2/9 (20)	0.471	
Child feeds self	2/21 (9.5)	0/28 (0)	0.179	
The caregiver interacts during mealtime	18/22 (81)	23/26 (88.5)	0.687	
The child receives altered food and/or liquid textures	9/22 (40.9)	25/28 (89.3)	0.001	
The caregiver is responsive to hunger cues and fullness cues	4/16 (25)	10/22 (45)	0.309	
The child does not cough when consuming liquids	2/8 (25)	3/12 (25)	1	
The meal include all of the five food groups	1/14 (7.1)	3/21 (14.3)	0.635	
The spoon or size of food offered is appropriate	2/15 (13)	16/22 (72)	0.001	
The caregiver allows ample time for the child to appropriately and safely eat/swallow each bite	No observations completed	3/6 (50)	‐‐	
The caregiver appropriately feeds the child at the child's pace	7/9 (80)	4/12 (40)	0.080	
The caregiver cleans/assists with cleaning children's hands after mealtime (yes = desired)	1/6 (16)	5/8 (62.5)	0.138	

## 4 DISCUSSION

This study explored the feeding practices, behaviours, difficulties and outcomes among children living within IBC. Key findings from this study indicate that feeding difficulties were common among children living within IBC with the most common being difficulty self‐feeding. Disability was a major factor underlying this challenge, with children having an increased risk of feeding difficulties if a disability is present. Overtime in the CNP, some feeding difficulties resolve for those with and without disabilities, although many children continue to experience feeding difficulties. Suboptimal feeding practices were observed, such as poor positioning, limited handwashing and inappropriate pacing of meals. These findings have rarely been described in this population and might explain the increased prevalence of malnutrition in this population (DeLacey et al., 2020; DeLacey et al., 2021; Ernst et al., 2021).

### 4.1 Feeding difficulties

Oral feeding is an important component of children's nutritional growth and development. When feeding difficulties are present it can limit physical, behavioural and cognitive development, increase risks for illness, disease and cause or exacerbate existing disabilities (Benjasuwantep et al., 2013; DeLacey et al., 2020; DeLacey et al., 2021; Ernst et al., 2021; Reif et al., 1995). Providing support for children with feeding difficulties at their baseline screening should be prioritised (Manikam & Perman, 2000; Reif et al., 1995). By addressing feeding issues early and effectively with training and resources for caregivers, long‐term feeding difficulties, malnutrition and delayed development could be minimised or avoided (Perry, 2005). Over 40% of those with feeding difficulties did not have a disability and are still at risk of becoming malnourished, even though not having a disability may not make their risk as obvious. Notably, many children saw their feeding difficulties resolve after 1 year in the CNP, 54/163 (33.1%) for children with disabilities and 57/106 (53.8%) for children without disabilities. It is likely that the programme had an impact on improving the feeding of children, even though some feeding difficulties resolve with age.

Moreover, the impact of how children are fed can lead to long‐term positive or negative associations with feeding (Reif et al., 1995). Diagnostic and Statistical Manual of Mental Disorders (DSM‐5) diagnosis for paediatric feeding disorders indicates that children can be experiencing fear and pain during the feeding process and this could lead to negative associations with mealtimes (Perry, 2005; American Psychiatric Association, 2016). However, caregivers often work long hours, receive very little training, maintain social‐emotional detachment and interaction is not considered a key function of their roles (The St. Petersburg‐USA Orphanage Research Team, 2005). Limited staffing and support can lead to limited time to engage and fully support each child (The St. Petersburg‐USA Orphanage Research Team, 2005).

### 4.2 Feeding practices

Mealtimes can be opportunities for positive interactive learning or stressful events with suboptimal feeding practices (G. A. Silva et al., 2016; The St. Petersburg‐USA Orphanage Research Team, 2005). Mealtimes are important because modelling desired behaviours by caregivers can teach children about eating practices or contexts of meals (Birch & Doub, 2014). What children learn during mealtimes from caregivers has an impact on their lifelong eating habits, nutritional status, cognitive and social development (Richter et al., 2019). Learning new feeding skills from peers or other children may also be limited because children in IBC are typically grouped by disability status or age regardless of developmental level or needs (Perry, 2005; The St. Petersburg‐USA Orphanage Research Team, 2005, 2008). Quantitative and qualitative data from the behaviour observations in our study indicate that caregivers need on‐going support to carry out optimal feeding for infants, young children and for those with disabilities.

Interactions during mealtimes varied widely between caregivers and sites—from no interaction to highly engaged. Positive interaction is essential for children's development and positive relationships can mitigate children's trauma (Perry, 2005). Despite this, suboptimal feeding practices and limited response to feeding cues, especially for infants and children with disabilities, were commonly noted. The St. Petersburg‐USA Orphanage Research Team findings that feeding regimes were often limited in interaction, with bottle propping, scraping of children's faces, refeeding of spilled food back into the child's mouth and children fed lying down were prevalent practices in all observation sites in this study (The St. Petersburg‐USA Orphanage Research Team, 2005, 2008). Additional poor feeding practices in our study included inappropriate pacing, positioning, limited interaction, forced feeding, lack of awareness of feeding cues, limited opportunities for self‐feeding and skill advancement, restrictive feeding schedules and limited offering of fluids. The pace of meals being fed to children was often reported to be rapid, and similar to the findings by The St. Petersburg‐USA Orphanage study which observed rapid feeding, with some children receiving as many as 30 spoonfuls per minute (Reilly et al., 1996; The St. Petersburg‐USA Orphanage Research Team, 2008).

Additionally, poor hygiene and sanitation practices were prevalent and should be addressed as a preventable route for illness and malnutrition. Specifically, handwashing was not frequently observed among caregivers or children. Other concerning feeding practices included feeding children cereal in their bottles and cutting bottle nipples to increase flow rate, which can increase the risk of aspiration as well as reduce nutrient intake. Also, inappropriate feeding utensils, such as spoons too large for children's mouths, poor seating options or inappropriate nipples and bottles for premature infants were noted. Some observed feeding practices were positive, such as the use of altered textures for food and liquids for children with disabilities, positive interaction during mealtimes, improved positioning, changes to serving sizes and appropriate environments for mealtimes but this varied by the feeder and site. Support for positive practices, such as good positioning, adequate fluid and dietary intake, food texture modifications, adaptive equipment and environmental modifications should be prioritised (Reilly et al., 1996).

### 4.3 Children with disabilities

We found disability status to be strongly related to feeding difficulties. Compared with children without disabilities, those with disabilities had a higher prevalence of feeding difficulties at their baseline and 1‐year screening. Children with disabilities had more than five times the risk, in adjusted analysis, of having a feeding difficulty at their baseline screening compared to children without disabilities. Feeding difficulties, such as difficulty self‐feeding, chewing, drinking from a cup and sucking, in addition to coughing or choking and difficulty swallowing were higher for children with disabilities. Similarly, Kuper and co‐workers, found that children with disabilities were more likely to experience feeding difficulties compared to their neighbours (OR = 1.9, 95% CI: 1.2–3.1) and are more likely to have difficulty self‐feeding (Kuper et al., 2015). Many children with disabilities have challenges feeding themselves or eating (DeLacey et al., 2020; Groce et al., 2014; Hume‐Nixon & Kuper, 2018). Teaching children with disabilities to feed themselves often takes additional time, resources and is often not done. This is a lost opportunity. Allowing additional time and resources as needed to teach these children to feed themselves, will create greater self‐efficacy, increase social participation and independence for the rest of their lives. It should be considered a long‐term investment in their futures (Groce et al., 2014; Hume‐Nixon & Kuper, 2018; Reilly, 1996).

Screening for feeding difficulties early could help with the identification of children who need additional support and feeding interventions to enable safe mealtimes and support growth (S. Johnson et al., 2016; Manikam & Perman, 2000). Identifying feeding difficulties could point to underlying oral‐motor problems related to neurological immaturity, delays or disabilities, which result in poor developmental outcomes (S. Johnson et al., 2016; Manikam & Perman, 2000).

Children with disabilities in IBC are at increased risk of malnutrition for a variety of reasons (DeLacey et al., 2020; DeLacey et al., 2021; Ernst et al., 2021). Poor fluid and dietary intake were noted for this group, which could lead to dehydration, malnutrition, feeding difficulties and illnesses (DeLacey et al., 2021).

### 4.4 Illnesses, supplementation and anthropometry

Children within IBC were commonly found to have been ill within the last month in IBC, with children with disabilities having a higher proportion of illnesses compared to those without a disability. The most common illnesses reported were a cough or cold (722/3335 [21.6%]). This could be related to a number of factors, including poor hygiene, inadequate dietary intake and other suboptimal feeding practices putting them at increased risk for illness (Victora et al., 2021). Anaemia is common in this population (DeLacey et al., 2021; Ernst et al., 2021). Frequent illnesses or anaemia can have consequences for children's development and impact brain functioning (Black et al., 2013; Victora et al., 2021). Notably, anaemia resolves for many children after 1 year in CNP, likely related in part to screening and treatment components of the programme. In this population, supplementation was common with nearly half of all children receiving a supplement at baseline. Mineral, vitamin and food supplementation was more prevalent among children with disabilities. This could raise a concern that the challenge of feeding children with disabilities is resulting in them being given supplements in lieu of teaching caregivers or children themselves feeding skills. Chronic poor dietary intake, frequent illnesses, micronutrient deficiencies and feeding practices could lead to poor growth. Children with disabilities are more likely to have lower anthropometric measurements compared to siblings and peers without disabilities (DeLacey et al., 2020; DeLacey et al., 2021; Ernst et al., 2021; Myatt et al., 2018). Our paper found nearly 30% of children with disabilities to have a low BMI, which may be related to the third of children who presented feeding difficulties (DeLacey et al., 2020; DeLacey et al., 2021; Ernst et al., 2021; Kuper et al., 2015).

### 4.5 Limitations

There were some limitations in our study. Although this study was from a large multicountry sample, this sample may not be representative of all IBC facilities in these countries since data were collected only from those participating in Holt International's CNP. Holt partnerships provide resources that many other institutions may not regularly have access to, including organisational and financial support for education, healthcare, nutrition and other child welfare needs.

Additionally, when analysing these data it is important to consider that some children's first screening was their first day in IBC, and for others, their first screening occurred after multiple years in IBC. Time in IBC for children still in care is censored at the final data pull date. Changes in feeding practices vary based on children's age, skill level and how long they are in IBC. Holt's CNP provides definitions and training around age‐ and disability‐appropriate feeding practices but we acknowledge that perceptions of child needs and abilities may have an element of subjectivity. For example, some Holt feeding specialists and trainers are from Western backgrounds/training and details of appropriate feeding practices vary by age or culture (e.g., although self‐feeding is an important part of development, in many cultures, caregivers feeding children is a sign of care). More objective tools could be used in the future.

Furthermore, disabilities were diagnosed by specialists within the countries but not all countries or specialists diagnose disabilities the same way. In the future, a standardised diagnostic tool could be used for more comparative analysis. However, grouping children, both those without disabilities and those with disabilities, do not fully address the individual needs of children. Children with some types of disabilities may be small or underweight for age based on clinical sequelae related to their specific disability. These disabilities may impede their ability to self‐feed, manipulate food in the mouth, safely swallow, digest food or be associated with conditions that would reflect in lower height or weight. Additionally, there are potentially unobserved variables that might confound the relationship observed, such as prenatal substance exposure, which could be related to both disability status and feeding difficulties that we were unable to include in the analysis. Finally, change in sample sizes over time and missing data could impact these interpretations. For example from the original data set, there was more missing data at 1 year of both children with and without disabilities who did not have a feeding problem. This could indicate that those who have fewer difficulties may be able to be placed into family‐based care more easily than those with feeding difficulties. Survival bias may also be present considering those who are sicker or with more severe disabilities that impact their ability to eat, may not live as long. Therefore results should be taken with some caution as the population of children with baseline to 1‐year screenings may differ from those who stay in IBC the longest and the overall effect of these biases is unknown. Future prospective studies may help understand their relative effects.

### 4.6 Recommendations

In light of many global issues, such as food insecurity, climate change and the COVID‐19 pandemic, the risk to vulnerable children is heightened, as is the risk of abandonment (Goldman et al., 2020; Headey et al., 2020; Victora et al., 2021). With the global goal of deinstitutionalizing children and strengthening families, addressing the needs of children and their caregivers, especially those with disabilities, is essential (DeLacey et al., 2021; Ernst et al., 2021; Goldman et al., 2020; Headey et al., 2020; Manikam & Perman, 2000; United Nations Human Rights Office of the High Commissioner, 1990; Victora et al., 2021). It is important to consider how to support and strengthen individual caregivers and families in communities who may lack the support, supervision and resources present in IBC (DeLacey et al., 2020; DeLacey et al., 2021; Ernst et al., 2021; Whetten et al., 2014). Future research needs to examine how best to support caregivers in different countries and cultures to provide high‐quality feeding practices, especially around quality interaction, children's feeding cues, pacing, satiety and feeding difficulties, such as aspiration (Perry, 2005; Reilly, 1996; Reilly et al., 1996). This could improve child health outcomes and nutritional status.

In light of how common feeding issues are, we recommend all caregivers who work in IBC receive training on child feeding and nutrition. Given the potential bias in this study, follow‐up with future cohorts prospectively would address some of the limitations in this paper and could focus on the needs of specific ages or those with or without disabilities as important subgroups. There could be more formal intervention research exploring the impact of feeding support programmes such as that run by Holt; more targeted research could also focus on specific elements of the programme, such as the use of Holt International's Feeding and Positioning Manual (Holt International et al., 2019).

## 5 CONCLUSION

As the global community works towards the deinstitutionalization of children, addressing the feeding needs of those living within IBC is a top priority. Poor feeding practices are common in IBC and can put children at risk for illnesses, malnutrition and can cause or exacerbate existing disabilities. Disabilities and feeding issues are strongly linked. Feeding and mealtimes offer not just the opportunity for good nutrition but are part of critical development and connections for children. Supporting each child's individual needs should be prioritised, with a focus on safe, positive and engaging meals. Caregivers play a critical role and should receive the resources to understand and provide support to children during mealtimes. Feeding regimes for all children living in IBC need to be routinely reviewed and evaluated; appropriate feeding for children with disabilities, in particular, needs to be carefully and consistently implemented. Based on the findings from this study, we believe this is a critically important and currently largely overlooked component of improving the health and well‐being of millions of children currently living in IBC.

## CONFLICTS OF INTEREST

The authors declare no conflicts of interest.

## ETHICS STATEMENT

London School of Hygiene and Tropical Medicine's Ethics Committee (ref: 22822). This study did not involve patients or the public in its development. We intend to disseminate this study through open access publication to the public and all stakeholders. Holt International has given consent for the publication.

## AUTHOR CONTRIBUTIONS

Emily DeLacey, Marko Kerac, Elizabeth Allen and Cally Tann designed the study. Emily DeLacey, Marko Kerac, Tracey Smythe, Michael Quiring, Cally Tann, Nora Groce, Elizabeth Allen and Evan Hilberg contributed to specific areas of the methods, data analysis, statistics, and quality control. Emily DeLacey, Marko Kerac, Evan Hilberg and Michael Quiring had access and verified the data. Emily DeLacey led the data analysis and wrote the first draft of the manuscript. Emily DeLacey, Marko Kerac, Michael Quiring, Cally Tann, Nora Groce, Elizabeth Allen, Tracy Kaplan, Erin Kaui, Rachael Catt, Raeanne Miller and Evan Hilberg contributed to the writing of the manuscript and agree with the manuscript's results and conclusions. All of the authors have read and approved the submitted manuscript.

## Data Availability

Data are available upon request after approval process from Holt International. STATA code available upon request from Holt International.

## APPENDIX A

**Table A1:** Full Table 2: Description of feeding practices and health variables of children living within institution‐based care in six countries at baseline and 1‐year screening

Feeding profile	All children at baseline	All children at 1 year	Children without disabilities at baseline	Children without disabilities at 1 year	Children with disabilities at baseline	Children with disabilities at 1 year
Feeding method, *n* (%)^a^	*N* = 3335	*N* = 1909	*N* = 2578	*N* = 1385	*N* = 757	*N* = 524
Fed with bottle	1398 (41.9)	525 (27.5)	1028 (39.9)	327 (23.6)	370 (48.9)	198 (37.8)
Selfâ€fed	1727 (51.8)	1046 (54.8)	1469 (57)	830 (59.9)	258 (34.1)	216 (41.2)
Fed with cup	930 (27.9)	650 (34.1)	804 (31.2)	504 (36.4)	126 (16.6)	146 (27.9)
Spoon fed	1,123 (33.7)	957 (50.1)	811 (31.5)	628 (45.3)	312 (41.2)	329 (62.8)
Tube fed	0	0	0	0	0	0
Fed with adaptive utensils	32 (1.0)	21 (1.1)	15 (.6)	2 (0.1)	17 (2.3)	19 (3.6)
Breastfed	9 (0.3)	1 (0.1)	7 (0.3)	1 (0.1)	2 (0.3)	0
Feed type, *n* (%)^a^						
Formula	1488 (44.6)	467 (24.5)	1108 (43)	316 (22.8)	380 (50.2)	151 (28.8)
Solid foods	1993 (59.8)	1314 (68.8)	1578 (61.2)	951 (68.7)	415 (54.8)	363 (69.3)
Animal milk	803 (24.1)	584 (30.6)	659 (25.6)	402 (29.0)	144 (19.0)	182 (34.7)
Rice cereal	445 (13.3)	534 (28)	306 (11.9)	339 (24.5)	139 (18.4)	195 (37.2)
Breast milk	11 (0.3)	1 (0.1)	9 (0.4)	1 (0.1)	2 (0.3)	0
Special diet	77 (2.3)	53 (2.8)	38 (1.5)	19 (1.4)	39 (5.2)	34 (6.5)
Feeding difficulty, *n* (%)						
Feeding issue present	378 (11.3)	243 (12.7)	153 (5.9)	83 (6.0)	225 (29.7)	160 (30.5)
Aspiration	14 (0.4)	11 (0.6)	0	2 (0.1)	14 (1.9)	9 (1.7)
Difficulty sucking	27 (0.8)	16 (0.8)	4 (0.2)	0	23 (3.0)	16 (3.1)
Cough/chokes during feeding	57 (1.7)	25 (1.3)	17 (0.7)	0	40 (5.3)	25 (4.8)
Difficulty feeding self (>1 year)	119 (3.6)	103 (5.4)	8 (0.3)	18 (1.3)	111 (14.7)	85 (16.2)
Reflux/heartburn	6 (0.2)	5 (0.3)	2 (0.1)	0	4 (0.5)	5 (1.0)
Poor appetite	111 (3.3)	82 (4.3)	72 (2.8)	47 (3.4)	39 (5.2)	35 (6.7)
Frequent vomiting/spitting up	19 (0.6)	8 (0.4)	7 (0.3)	1 (0.1)	12 (1.6)	7 (1.3)
Difficulty drinking from a cup (>1 year)	53 (1.6)	43 (2.3)	2 (0.1)	3 (0.2)	51 (6.7)	40 (7.6)
Difficulty swallowing	63 (1.9)	50 (2.6)	3 (0.1)	0	60 (7.9)	50 (9.5)
Difficulty chewing	91 (2.7)	82 (4.3)	8 (0.3)	1 (0.1)	83 (11.0)	81 (15.5)
Picky eater	69 (2.1)	44 (2.3)	35 (1.4)	19 (1.4)	34 (4.5)	25 (4.8)
Food allergy/intolerance	14 (0.4)	8 (0.4)	10 (0.4)	5 (0.4)	4 (0.5)	3 (0.6)
Bad teeth (> 1 year)	22 (0.7)	24 (1.3)	9 (0.4)	4 (0.3)	13 (1.7)	20 (3.8)
Other	5 (0.2)	1 (0.1)	2 (0.1)	0	3 (0.4)	1 (0.2)
Supplements, *n* (%)^a^						
Currently taking food supplements	225 (6.8)	42 (2.2)	157 (6.1)	15 (1.1)	68 (9.0)	27 (5.2)
Currently taking mineral/vitamin supplements	1626 (48.8)	847 (44.4)	1176 (45.6)	572 (41.3)	450 (59.5)	275 (52.5)
Complete multivitamin	219 (6.6)	232 (12.2)	164 (6.4)	177 (12.8)	55 (7.3)	55 (10.5)
Vitamin A	231 (6.9)	192 (10.1)	148 (5.7)	123 (8.9)	83 (11)	69 (13.2)
Vitamin B_12_	191 (5.7)	123 (6.4)	132 (5.1)	64 (4.6)	59 (7.8)	59 (11.3)
Zinc	206 (6.2)	89 (4.7)	168 (6.5)	56 (4.0)	38 (5.0)	33 (6.3)
Lysine	30 (0.9)	15 (0.8)	30 (1.2)	14 (1.0)	0	1 (0.2)
Iron	271 (8.1)	109 (5.7)	242 (9.4)	83 (6.0)	29 (3.8)	26 (27.1)
Vitamin C	960 (28.8)	517 (27.1)	733 (28.4)	324 (23.4)	227 (30.0)	193 (36.8)
Vitamin B complex	247 (7.4)	146 (7.7)	166 (6.4)	72 (5.2)	81 (10.7)	74 (14.1)
Calcium	801 (24.0)	213 (11.2)	542 (21.0)	117 (8.5)	259 (34.2)	96 (18.3)
Fish oil/omega 3/EPA/DHA	1 (0.03)	3 (0.2)	0	3 (0.2)	1 (0.03)	0
Vitamin D	775 (23.2)	264 (13.8)	512 (19.9)	161 (11.6)	263 (34.7)	103 (19.7)
Folate	102 (3.1)	101 (5.3)	84 (3.3)	49 (3.5)	18 (2.4)	52 (9.9)
Probiotics	12 (0.4)	5 (0.3)	12 (0.5)	5 (0.4)	0	0
Other	238 (7.1)	147 (7.7)	196 (7.6)	104 (7.5)	42 (5.6)	43 (8.2)
Illnesses/symptoms, *n* (%)^a^						
Fever	438 (13.1)	193 (10.1)	295 (11.4)	121 (8.7)	143 (18.9)	72 (13.7)
Constipation	40 (1.2)	13 (0.7)	22 (0.9)	0	18 (2.4)	13 (2.5)
Diarrhoea	172 (5.2)	40 (2.1)	116 (4.5)	23 (1.7)	56 (7.4)	17 (3.2)
Nausea/vomiting	163 (4.9)	32 (1.7)	111 (4.3)	20 (1.5)	52 (6.9)	12 (2.3)
Cough/cold	722 (21.6)	395 (20.7)	489 (19.0)	222 (16.2)	233 (30.8)	173 (33.0)
Hospital	135 (4.0)	45 (2.4)	64 (2.5)	26 (1.9)	71 (9.4)	19 (3.6)
Anaemia status, *n* (%)	*N* = 2828	*N* = 1511	*N* = 2167	*N* = 1101	*N* = 661	*N* = 410
None	2065 (73.0)	1314 (87.0)	1604 (74.0)	969 (88.0)	461 (69.7)	345 (84.2)
Mild	438 (15.5)	136 (9.0)	346 (16.0)	102 (9.3)	92 (13.9)	34 (8.3)
Moderate	307 (10.9)	59 (3.9)	212 (9.8)	30 (2.7)	95 (14.4)	29 (7.1)
Severe	18 (0.6)	2 (0.1)	5 (0.2)	0	13 (2.0)	2 (0.5)
Body mass index for age *z*‐score, *n* (%)	*N *= 3113	*N* = 1790	*N* = 2408	*N* = 1312	*N* = 705	*N* = 478
Risk of overweight or obesity (>+1 SD to ≤5)	361 (11.6)	226 (12.6)	286 (11.9)	172 (13.1)	75 (10.6)	54 (11.3)
Normal weight (−2 to +1 SD)	2305 (74.0)	1404 (78.4)	1882 (78.2)	1051 (80.1)	423 (60.0)	353 (73.9)
Thin/underweight (<−2 to −3 SD)	291 (9.4)	119 (6.7)	175 (7.3)	73 (5.6)	116 (16.5)	46 (9.6)
Severe thinness/underweight (<−3 to ≥−5 SD)	156 (5.0)	41 (2.3)	65 (2.7)	16 (1.2)	91 (12.9)	25 (5.2)

^a^ Not mutually exclusive variables.

**Table A2:** Young children feeding behaviour observations at baseline and evaluation

Young children feeding behaviour observations				
	15 observations	32 observations	Fisher's exact (two‐sided) *p*‐value	Qualitative summary
	Baseline	Evaluation		Length of feeding: 5–30 min
	*n*/*N* (%)	*n*/*N* (%)		
Cleans hands before feeding using hand sanitiser or hot, soapy water	4/10 (40)	8/15 (53)	0.688	Limited handwashing observed; feeding on only organization's schedule; feeding cues not observed by caregivers; multiple children fed at the same time; force feeding; limited self‐feeding; caregivers are attentive to children; environments were calm, quiet and appropriately lit
Cleans/assists with cleaning young children's hands before mealtime	4/11 (36)	6/14 (42.9)	1	
Caregiver does not leave young children unattended during mealtimes	14/14 (100)	29/31 (93.5)	1	
Caregiver supervises and assists young children (<3 years) with using a spoon or cup	11/12 (91.6)	15/24 (62.5)	0.115	
Caregiver allows older children (>3 years) to feed themselves with minimal assistance	10/11 (90)	12/14 (85.7)	1	
Caregiver feeds when the child is showing signs of hunger	8/9 (80)	22/27 (81.4)	1	
Caregiver stops feeding infants when showing signs of fullness	11/12 (91.6)	25/29 (86.2)	1	
Caregiver allows young children to decide how much they will eat	6/12 (50)	5/17 (29.4)	0.438	
Caregiver feeds one child at a time	6/12 (50)	10/21 (47.6)	1	
Caregiver does not allow multiple children to use the same spoon, cup or dish	5/6 (83)	9/14 (64.3)	0.613	
Caregiver cleans hands after mealtime using hand sanitiser or hot, soapy water	6/10 (60)	10/14 (71.4)	0.673	
Caregiver cleans/assists with cleaning young children's hands after mealtime	6/11 (54)	16/22 (72)	0.437	
The caregiver allows ample time for the child to appropriately and safely eat/swallow each bite	No observations completed	3/6 (50)	‐	
The caregiver appropriately feeds the child at the child's pace	7/9 (80)	4/12 (40)	0.080	
The caregiver cleans/assists with cleaning children's hands after mealtime (yes = desired)	1/6 (16)	5/8 (62.5)	0.138	

**Table A3:** Infants bottle‐feeding behaviour observations at baseline and evaluation

Bottle‐feeding behaviour observations				
	11 observations	33 observations	Fisher's exact (two‐sided) *p*‐value	Qualitative summary
	Baseline	Evaluation		Length of feeding: 5–30 min
	*n*/*N* (%)	*n*/*N* (%)		
Caregiver cleans hands before feeding using hand sanitiser or hot, soapy water	0/4 (0)	5/19 (26.3)	0.539	Limited handwashing observed; frequent bottle propping of infants; frequent cut nipples on bottles; cereal or porridge added to bottles; limited adaptive bottles or bottles for premature infants; feeding on only schedule; hunger cues not observed by caregivers; multiple children fed at the same time; limited or no burping observed and children laid down after meals; environments were calm, quiet and appropriately lit
Caregiver properly positions infant in a semi‐upright/upright position in their arms for feeding	5/8 (45.5)	14/19 (73.7)	0.658	
Caregiver offers only formula or milk in the bottle	9/11 (81)	30/33 (90)	0.586	
Caregiver does not mix rice cereal and formula in the bottle	7/10 (70)	26/29 (89.7)	0.049	
Caregiver does not cut large holes in bottle nipples	7/10 (70)	25/29 (86.2)	3.44	
Caregiver uses adaptive bottles when appropriate	2/6 (30)	4/11 (36)	1	
Caregiver feeds infant on demand	9/10 (90)	7/16 (43.8)	0.037	
Caregiver checks milk temperature before feeding an infant	6/7 (85.7)	13/22 (59)	0.367	
Caregiver feeds one infant at a time	8/11 (72)	24/31 (77.4)	1	
Caregiver does not share bottle or formula among multiple infants	8/10 (80)	26/26 (100)	0.071	
Caregiver burps infant before lying down or keeps an infant in a semi‐upright/upright position for at least 15 min following feeding	6/8 (75)	11/27 (40.7)	0.121	
Caregiver cleans hands after feeding using hand sanitiser or hot, soapy water	2/5 (40)	1/15 (6)	0.140	

STROBE Statement—Checklist of items that should be included in reports of cohort studies (von Elm et al., 2007)

**Table:**

	Item no	Recommendation	Page #
Title and abstract	1	(a) Indicate the study's design with a commonly used term in the title or the abstract	1
		(b) Provide in the abstract an informative and balanced summary of what was done and what was found	3
Introduction			
Background/rationale	2	Explain the scientific background and rationale for the investigation being reported	5
Objectives	3	State‐specific objectives, including any pre‐specified hypotheses	6
Methods			
Study design	4	Present key elements of study design early in the paper	6
Setting	5	Describe the setting, locations, and relevant dates, including periods of recruitment, exposure, follow‐up and data collection	7
Participants	6	(a) Give the eligibility criteria, and the sources and methods of selection of participants. Describe methods of follow‐up	8
		(b) For matched studies, give matching criteria and number of exposed and unexposed	‐
Variables	7	Clearly define all outcomes, exposures, predictors, potential confounders, and effect modifiers. Give diagnostic criteria, if applicable	8
Data sources/measurement	8^a^	For each variable of interest, give sources of data and details of methods of assessment (measurement). Describe comparability of assessment methods if there is more than one group	8
Bias	9	Describe any efforts to address potential sources of bias	17
Study size	10	Explain how the study size was arrived at	Figure 1
Quantitative variables	11	Explain how quantitative variables were handled in the analyses. If applicable, describe which groupings were chosen and why	8
Statistical methods	12	(a) Describe all statistical methods, including those used to control for confounding	8
		(b) Describe any methods used to examine subgroups and interactions	8
		(c) Explain how missing data were addressed	8/17
		(d) If applicable, explain how loss to follow‐up was addressed	8
		(e) Describe any sensitivity analyses	‐
Results			
Participants	13^a^	(a) Report numbers of individuals at each stage of study—for example, numbers potentially eligible, examined for eligibility, confirmed eligible, included in the study, completing follow‐up, and analysed	10/Figure 1
		(b) Give reasons for nonparticipation at each stage	Figure 1
		(c) Consider use of a flow diagram	Figure 1
Descriptive data	14^a^	(a) Give characteristics of study participants (e.g., demographic, clinical and social) and information on exposures and potential confounders	10
		(b) Indicate number of participants with missing data for each variable of interest	Noted in each table
		(c) Summarise follow‐up time (e.g., average and total amount)	13
Outcome data	15^a^	Report numbers of outcome events or summary measures over time	10–14
Main results	16	(a) Give unadjusted estimates and, if applicable, confounder‐adjusted estimates and their precision (e.g., 95% confidence interval). Make clear which confounders were adjusted for and why they were included	10–14
		(b) Report category boundaries when continuous variables were categorised	10
		(c) If relevant, consider translating estimates of relative risk into absolute risk for a meaningful time period	‐
Other analyses	17	Report other analyses done—for example, analyses of subgroups and interactions, and sensitivity analyses	10–14
Discussion			
Key results	18	Summarise key results with reference to study objectives	15
Limitations	19	Discuss limitations of the study, taking into account sources of potential bias or imprecision. Discuss both direction and magnitude of any potential bias	15–17
Interpretation	20	Give a cautious overall interpretation of results considering objectives, limitations, multiplicity of analyses, results from similar studies and other relevant evidence	15–17
Generalisability	21	Discuss the generalisability (external validity) of the study results	15–19
Other information			
Funding	22	Give the source of funding and the role of the funders for the present study and, if applicable, for the original study on which the present article is based	2
